# A Rare Case of Carotid Web Presenting with Ischemic Stroke in a Young Woman and a Brief Review of the Literature

**DOI:** 10.1155/2018/3195679

**Published:** 2018-02-19

**Authors:** Kyaw Kyaw, Htun Latt, Sammy San Myint Aung, Jay Babu, Rajesh Rangaswamy

**Affiliations:** ^1^Institute for Heart and Vascular Health, Renown Regional Medical Center, 1500 E. 2nd St. No. 302, Reno, NV 89502, USA; ^2^Department of Internal Medicine, University of Nevada, Reno School of Medicine, 1155 Mill St. No. W11, Reno, NV 89502, USA; ^3^Department of Biology, University of Nevada, Reno, 1664 N. Virginia Street, Reno, NV 89557, USA; ^4^Department of Radiology, Renown Regional Medical Center, 1155 Mill St., Reno, NV 89502, USA

## Abstract

Carotid web is a radiological description of a shelf-like intraluminal filling defect in the carotid bulb. It is histologically defined as atypical fibromuscular dysplasia (FMD), with abnormal fibrosis and smooth muscle cell hyperplasia in the *tunica intima*. The spur-like intraluminal protrusion can serve as a nidus for thrombus formation, which could cause systemic embolism and ischemic strokes. We report a case of a 20-year-old female patient presenting with acute ischemic stroke in the ipsilateral middle cerebral artery (MCA) territory. We also discuss the incidence, the prevalence, the pathophysiology, the treatment, and the recurrence of carotid web based on the currently available literature.

## 1. Introduction

Carotid web is a radiological description of a shelf-like intraluminal filling defect in the carotid bulb [1–4]. The word “web” was firstly used by Momose and New to analyze the causes of nonatheromatous stenosis in the internal carotid artery (ICA) in 1973 [5]. Because of the extreme rarity and unfamiliarity of the disease, the carotid web was variously described in the literature as carotid bulb web, carotid bulb diaphragm, carotid bulb septa, pseudovalvular folds, atypical fibromuscular dysplasia, and atypical fibromuscular hyperplasia [6]. Carotid web is a very rare cause of ischemic stroke in young patients (age < 60 years old) with suspected stroke [1–4]. Here, we report a case of a 20-year-old female patient with left carotid web, who presented with acute ischemic stroke in ipsilateral middle cerebral artery (MCA) territory.

## 2. Case Description

**Figure 1:**
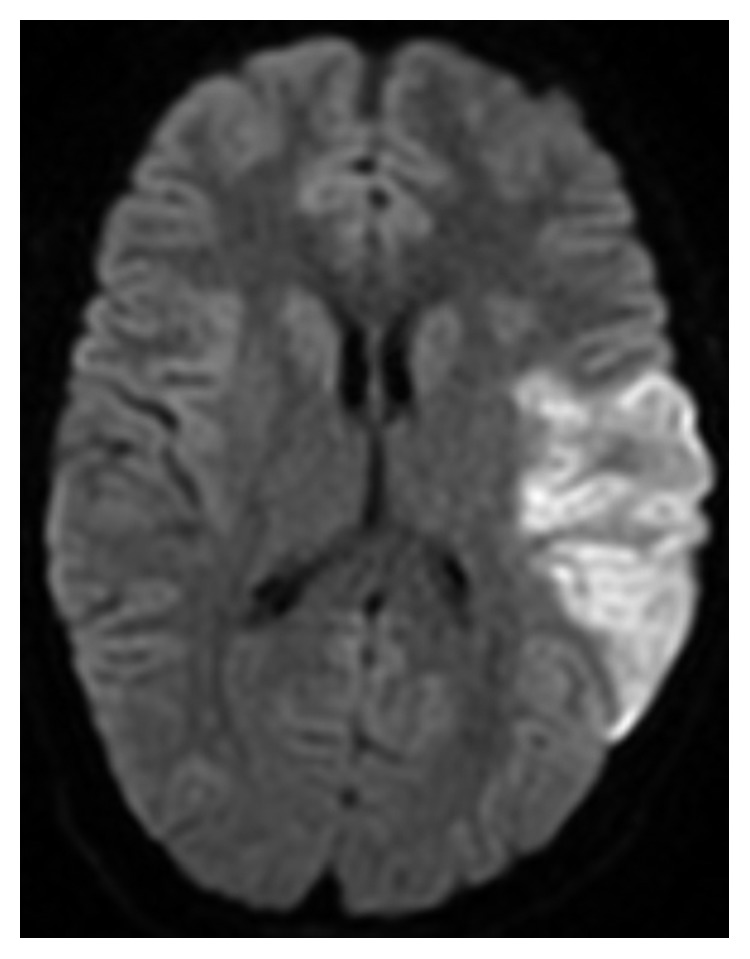
MRI brain without contrast (DWI—diffusion-weighted image) showing acute ischemic infarct in left temporoparietal lobe in the distribution of left MCA.

**Figure 2:**
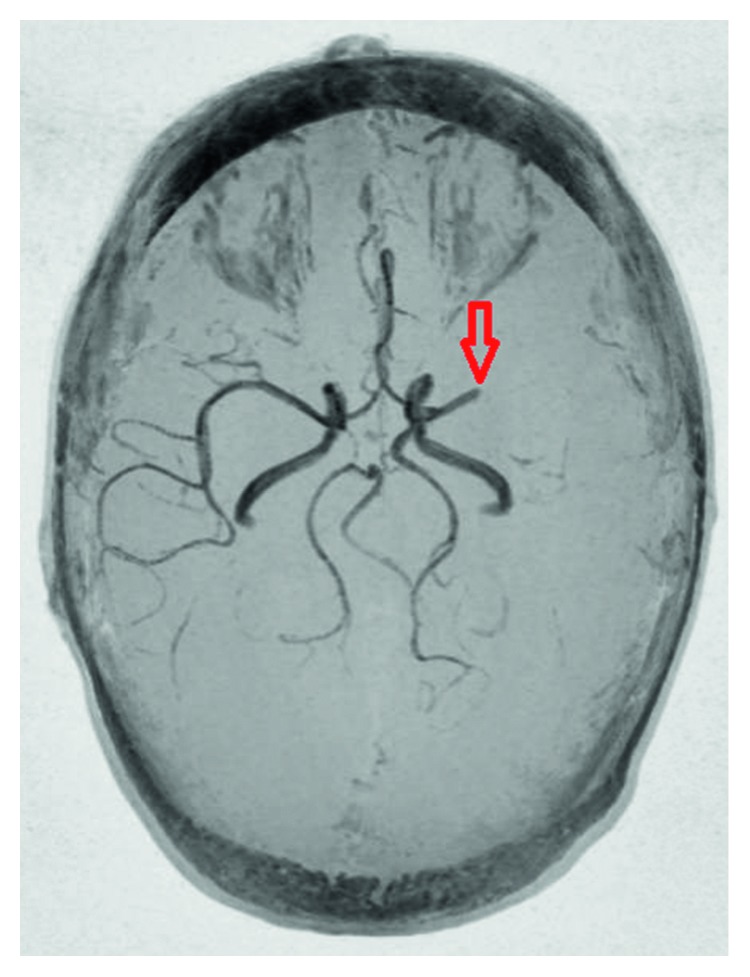
MRA brain showing total occlusion of M1 segment of left MCA (red arrow) and normal circle of Willis.

A 20-year-old Caucasian female with no known medical problem was transferred to our facility for left MCA stroke 8 hours after the new onset of aphasia. She initially presented to outside facility where magnetic resonance imaging of the brain (MRI brain) without contrast showed acute ischemic infarct involving the distribution of left MCA, approximately 9 cm in the area of left temporoparietal lobe (Figure 1). MR angiogram (MRA) showed total occlusion of M1 segment of left MCA (Figure 2). Her only medication was oral contraceptive pill, and she was a nonsmoker.

**Figure 3:**
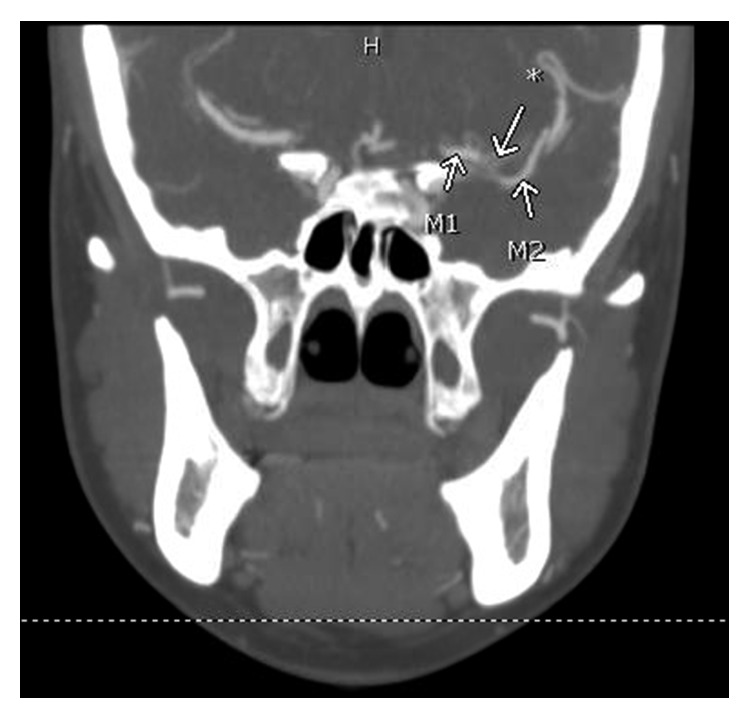
CTA neck showing a short segment of partially recanalized vessel (∗) between M1 and M2 segments of left MCA.

**Figure 4:**
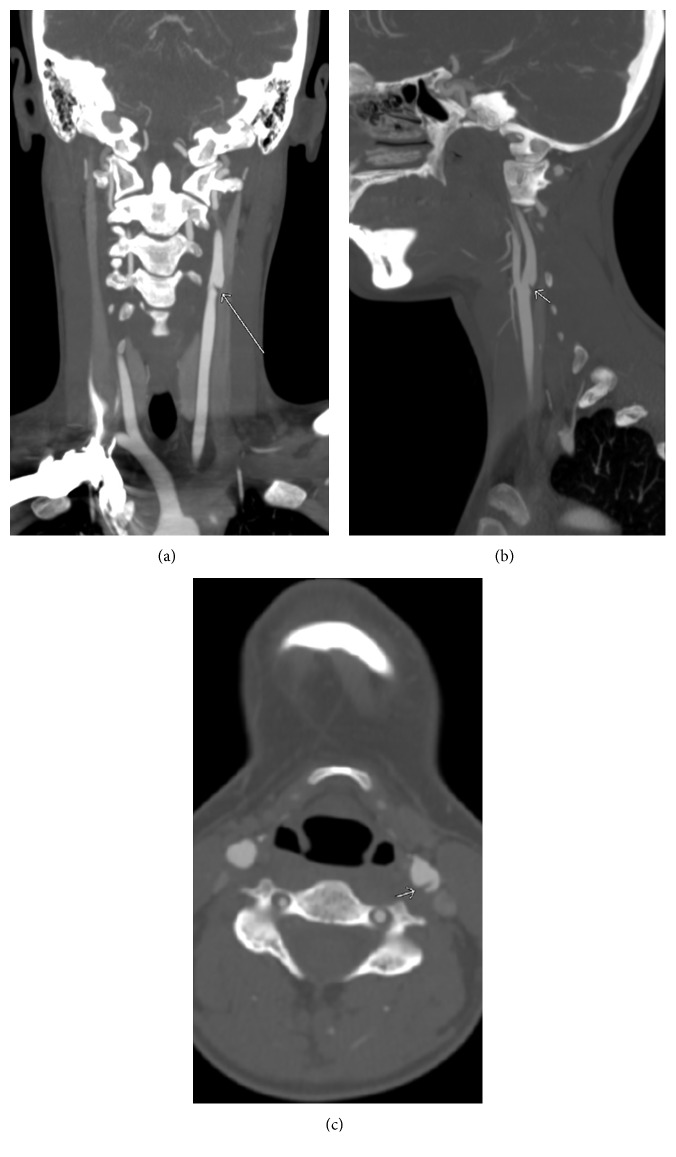
CTA neck showing carotid web in the left internal carotid artery (arrows).

At our facility, vital signs were stable. On examination, expressive aphasia and slight right-sided facial droop were noted. Other cranial nerves including facial sensation were intact. Gross motor and sensory functions, deep tendon reflexes, and Babinski reflexes were normal. NIH Stroke Scale/Score (NIHSS) was 6 (1B +2, aphasic; 1C +2, unable to perform tasks for blink eyes and squeeze hands; 10 +2, severe dysarthria). Neurology was immediately consulted. Aspirin suppository 300 mg was given rectally and shortly afterwards, and atorvastatin 80 mg was given through a nasogastric tube. However, no tissue plasminogen activator (tPA) was given since she was outside therapeutic window. Computed tomography angiogram (CTA) neck showed a short segment of partially recanalized vessel between M1 and M2 segments of left MCA (Figure 3). Also CTA neck revealed a short segment of linear filling defect in proximal left ICA, most consistent with a carotid web (Figure 4). Neurointerventionist and vascular surgeon were consulted, and both consultants deferred any invasive procedure because of acute cerebrovascular accident (CVA), large area of infarct, and partial recanalization of the previously thrombosed vessel as evidenced in CTA. No anticoagulation was given, but clopidogrel was added. She was conservatively treated with permissive hypertension. Twelve-lead resting electrocardiogram showed normal sinus rhythm.

Later, workup for hypercoagulability and vasculitis was done. Blood tests showed normal protein C and S and antithrombin III levels. Factor V Leiden, C-reactive protein, antinuclear antibody, pregnancy test, and lupus anticoagulant were negative. Transthoracic echocardiogram with saline (bubble) study was performed and showed no evidence of right to left shunt by agitated saline challenge. MR venogram was normal. She continued to have severe dysphagia, requiring feeding gastrostomy tube. No new or recurrent events were noted during the hospital stay. No abnormal rhythm was reported on cardiac monitor. She was discharged on conservative medical therapy with aspirin, clopidogrel, and atorvastatin.

## 3. Discussion

Carotid web is identified in angiogram as a linear spur-like intraluminal projection along the posterior wall of proximal internal carotid artery (ICA) on oblique sagittal section and as a septum on axial section [1, 2]. It recently has been referred as an atypical variant of fibromuscular dysplasia (FMD), with histological feature of abnormal fibrosis and smooth muscle cell hyperplasia in the tunica intima [3, 7–11]. Typical FMD has abnormal cell growth within the tunica media. Furthermore, typical FMD usually occurs in renal and carotid arteries of middle-age white women but is rarely symptomatic [7, 10, 11]. In contrast, the atypical FMD with abnormal intimal cell growth in the carotid web tends to extend into the arterial lumen and creates turbulent flow [1]. It serves as a nidus of thrombus formation and may cause ischemic stroke via arterial embolism [1–4].

Limited data are available for the epidemiology of carotid webs. We reviewed and summarized 4 recent case-control studies regarding the incidence and prevalence of carotid web. Joux et al. reported the incidence of carotid web with ipsilateral stroke was 3.8 (4.3 in women and 3.2 in men) per 100,000 person-year in young Afro-Caribbean population [2]. Choi et al. and Coutinho et al. estimated that the prevalence of carotid web was 1.0–1.2% in patients who were suspected of strokes and underwent CTA [1, 4]. And Sajedi et al. outlined that the prevalence of carotid web was 9.4–37% in stroke patients without identifiable cause as against 1–7% in nonstroke patients [2–4]. The mean age of symptomatic carotid web was 38.3–46.7 years old [2, 3]. Generally, the carotid web is more common in young women with strokes of unknown origin [1–4].

The etiology of carotid web is unknown. Some possible explanations are genetic propensity, chronic vascular injury, hormonal factors, and vasa vasorum abnormalities [9]. Oral contraceptive (OC) pills may cause arterial intimal hyperplasia in young women and could be a possible cause of carotid web [12, 13]. Carotid web may present with ischemic stroke in ipsilateral MCA territory. It is often diagnosed by CTA though it can be detected in sonogram or MRI [14–16]. Digital subtraction angiography (DSA) is the gold standard test for carotid web. The current treatment of carotid web includes carotid endarterectomy (CEA), endovascular stenting, and conservative medical therapy with antiplatelet or anticoagulant. In 2014, Joux et al. published 30% recurrent symptoms of carotid web in patients with antiplatelet therapy and no recurrent symptoms of carotid web in patients with surgical therapy [17]. To date, no large studies have reported the recurrent rate among the patients treated with endovascular stenting or anticoagulant therapy. CEA or endovascular stenting is preferred to prevent recurrence of stroke [3, 6, 8, 17].

Our 20-year-old Caucasian female patient initially presented with left MCA infarct as evidenced in MRI and MRA brain. Subsequently, a proximal left ICA carotid web was discovered in carotid CTA. Workup was negative for vasculitis, hypercoagulability, and right to left intracardiac shunt. And MRV of the brain was unremarkable. Because of the acute CVA, large infarct size, and partial recanalization of the previously thrombosed MCA as evidenced in CTA, no intervention was performed during this admission. Follow-up is planned in next 4 weeks for further management.

## 4. Conclusion

Carotid web, a radiological entity and an atypical FMD, is an exceptionally rare cause of acute ischemic stroke in young patients. Since its first description in 1973, the knowledge of the rare disease has been expanded. However, more studies are demanded for a better understanding of the epidemiology, pathophysiology, treatment, and prognosis of carotid web. Our case highlights the awareness of carotid web in young patients, who have ischemic stroke in MCA territory without any identifiable cause.
